# Topiramate for anger control: A systematic review

**DOI:** 10.4103/0253-7613.66834

**Published:** 2010-06

**Authors:** Bindu Susan Varghese, A. Rajeev, Mark Norrish, Saleh Bin Mohammed Al Khusaiby

**Affiliations:** Department of Medical Pharmacology, Oman Medical College, P.O. 391, P.C. 321, Al Tarif, Sohar, Sultanate of Oman; 1 Epidemiology, Oman Medical College, P.O. 391, P.C. 321, Al Tarif, Sohar, Sultanate of Oman; 2 Behavioral Medicine, Oman Medical College, P.O. 391, P.C. 321, Al Tarif, Sohar, Sultanate of Oman; 3 Pediatrics and Medical Ethics, Oman Medical College, P.O. 391, P.C. 321, Al Tarif, Sohar, Sultanate of Oman

**Keywords:** Aggression, anger control, topiramate

## Abstract

**Background:**

Uncontrolled anger while being most commonly associated with personality disorders could also be part of many other conditions such as chronic low back ache and post-traumatic stress disorder. The intensity of anger as an emotional state at a particular time is known as “State Anger,” whereas how often angry feelings are experienced over time is known as “Trait Anger.” Anger could also manifest as expression of anger toward other persons or objects in the environment (Anger-Out), holding in or suppressing angry feelings (Anger-In) and controlling angry feelings by preventing the expression of anger toward other persons or objects in the environment or controlling suppressed angry feelings by calming down or cooling off (Anger Control).

**Objective:**

To prove the effectiveness of topiramate in the control of anger as compared to placebo and to disprove that its use leads to psychiatric adverse events by systematically reviewing the available randomized controlled trials.

**Materials and Methods:**

The basic search was performed in MEDLINE (1966 through November 2008) combined with the optimal search strategy for randomized controlled trials described in the Cochrane Reviewers’ Handbook. To update this search, we regularly screened citations from PubMed till November 2008 for eligible studies or reviews that might include eligible studies. The Cochrane Central Register of Controlled Trials (CENTRAL) was searched using the terms “topiramate” and “anger or aggression.” In addition, we screened bibliographies of reviews and identified articles. Randomized clinical trials wherein study participants were aggressive adults were included.

**Results:**

We could arrive at a weighted mean difference of −3.16 (−3.64 to −2.68) in State Anger. The reduction in the score was highest in borderline personality disorder (BPD) patients as compared to those with low back ache. Trait Anger dropped by −2.93 (−3.49 to −2.37), especially in female BPD patients. Anger In reduced more or less uniformly across the studies by −1.43 (−1.84 to −1.03). Anger Out decreased by −2.8 (−3.19 to −2.42). This effect was minimal among the male BPD patients. Anger Control uniformly increased across the four studies by 2.32 (2.00−2.64). There is sufficient evidence to suggest that topiramate is significantly effective in stabilizing the “trait anger” while reducing the “state anger.” “Anger Out” and “hostility” were significantly reduced. “Anger In” was the feature that was the least affected, although this was significant. This suggests that topiramate is effective in controlling anger. There was no suggestion of topiramate precipitating psychomorbidity.

**Conclusions:**

Topiramate appears to be a safe and effective drug in the management of anger/aggression. Additional research is needed to determine whether these results can be reproduced and how long lasting are the benefits of long-term treatment with topiramate.

## Introduction

Uncontrolled anger, while being most commonly associated with personality disorders, could also be part of many other conditions such as chronic low back ache and post-traumatic stress disorder (PTSD). Anger can be considered as state anger, an episode of anger occurring at a specified time, and as trait anger, an aspect of personality,[1,2] and is typically measured on four State Anger, Trait Anger, Anger In, Anger Out and Anger Control (STAXI) scales.[3]

The prevalence of personality disorder in the general population is between 5.9 and 13%.[4] The prevalence of antisocial (dissocial) personality disorder has been shown to be about 2–3%[5] in the general population, while that of borderline personality disorder (BPD) is about 1.7–2%.[6] In a prison survey, 11% of the sentenced prisoners in the United Kingdom had a personality disorder.[7] PTSD is as common as 7.8% in terms of lifetime rate in the population.[8] Definition of uncontrolled anger comes very near, within ICD-10, to a group of disorders termed “Habit and impulse disorders,” i.e. “repeated acts that have no clear rational motivation and that generally harm the patient’s own interests and those of other people.” These particular disorders appear to have originated from an earlier concept of “episodic dyscontrol syndrome,” which was described by Mark and Ervin.[9] This syndrome is characterized by unprovoked aggressive outbursts, sometimes with amnesia for the outburst, and is associated with brain damage and pathological intoxication with alcohol. The authors considered that subclinical epileptic seizures might be underlying these sudden outbursts, and found electroencephalogram changes in their subject.

Following the above findings, antiepileptic drugs were used to reduce the frequency of aggressive outbursts, especially in those who had suffered brain injury, or where an organic basis for aggression was perceived to exist. Among the many different agents used, topiramate is one of the most commonly mentioned drug for the treatment of BPD.[10,11] It has been subjected to a number of placebo-controlled trials and is now sometimes used as an agent for treating diverse conditions such as neuroleptic-induced weight gain and epilepsy at its extreme, even in pediatric patients.[12,13] As is well known, anticonvulsants are generally structural analogues of gamma-amino butyric acid, and the psychotropic and other effects may be exerted by producing release of dopamine, norepinephrine and serotonin. Topiramate specifically blocks AMPA/Kainate-gated ion channels and sodium channels and positively modulates gamma butyric acid receptors.[14] However, there are suggestions in the literature that topiramate seems to behave paradoxically. When this is used as a primary antiepileptic, it has been claimed to precipitate psychomorbidity. But, when used in BPD, it seems to provide emotional benefits such as anger control. While topiramate is well tolerated in general, it is associated with a variety of adverse effects (acute myopia, angle closure glaucoma, urinary tract stones, cognition difficulties and more) when used for treating epilepsy, probably at higher doses. These observations are often made by case–series and not typically by a well-conducted randomized controlled trial. Anger and aggression are emotions that often have negative implications in social life and hence result in disruption of the orderly behavior of individuals or organizations. While successful individuals have in them a component of aggression that drives them to higher objectives, the negative side of aggression, i.e. anger, can destroy the ultimate goal that they have set out to achieve. We can cite a number of celebrities who have been the victims of this emotion and its after-effects. While the literature certainly indicates control of anger by using pharmacotherapy as well as psychotherapy as part of many of the treatment schedules, a cohesive analysis of only anger control as an endpoint in a randomized controlled trial or, still better, a metaanalysis, seems difficult to find.

Hence, we aimed to systematically review the available randomized and quasi-randomized controlled trials of topiramate for its specific action on anger control. We aimed to determine whether there is evidence to:

- prove that topiramate is effective in the control of anger as a symptom as compared to placebo or other therapies
- disprove observations of some uncontrolled series on epileptic patients that suggest that the use of topiramate leads to psychiatric adverse events such as anger.

## Materials and Methods

Randomized and quasi-randomized (using methods such as alternation) clinical trials were included in the review. Trials that did not make an explicit statement about the allocation method but were described as double-blind (referring to blinding of patients and blinding of recruiting, treating and evaluating staff) were included unless there were clear reasons to assume that allocation was not randomized.

Study participants were required to be aggressive adults. Trials including individual participants below 18 years of age were included provided that the mean age of the trial participants clearly indicated that the majority of the patients were adults (e.g., the age range was 16–61 years, with a mean age of 41 years). Trials conducted among patients who suffered from other types of aggression, including that in BPDs, were included. Included studies were required to have at least one arm in which topiramate was used. At least one of the following outcomes must have been measured (but not necessarily reported in sufficient detail) to allow effect size calculation:

- Four STAXI scales[3] – State Anger, Trait Anger, Anger Out, Anger Control – or any equivalent measure of component or global response. The State Anger scale assesses the intensity of anger as an emotional state at a particular time. The Trait Anger scale measures how often angry feelings are experienced over time. The Anger Expression and Anger Control scales assess relatively independent anger-related traits: (i) expression of anger toward other persons or objects in the environment (Anger-Out), (ii) holding in or suppressing angry feelings (Anger-In) and (iii) controlling angry feelings by preventing the expression of anger toward other persons or objects in the environment or controlling suppressed angry feelings by calming down or cooling off (Anger Control). Individuals rate themselves on the scales that assess both the intensity of their anger at a particular time and the frequency at which anger is experienced, expressed and controlled.
- Symptoms: a change in self-reported feelings of anger and impulsiveness, either an increase or decrease in the frequency and severity.
- Behavior: a reduction in aggression, either to self or others; a reduction in impulsiveness.

The basic search was performed in MEDLINE (1966 through November 2008) using the search terms “topiramate OR anticonvulsant” and “anger (exploded) OR aggression” combined with the optimal search strategy for randomized controlled trials described in the Cochrane Reviewers’ Handbook.[1] To update this search, we regularly screened citations from the search “anger AND topiramate” and also “aggression AND topiramate” in PubMed for eligible studies or reviews that might include eligible studies. The Cochrane Central Register of Controlled Trials (CENTRAL) was searched using the terms “topiramate” and “anger or aggression.” In addition, we screened bibliographies of reviews and identified articles.

**Figure 1:**
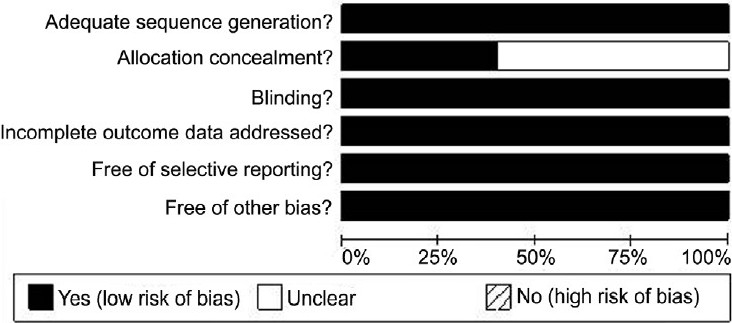
The fraction of studies fulfilling the various domains of “risk of bias analysis” as per the Cochrane Handbook method.

One reviewer screened titles and abstracts of all references identified and excluded all citations that were clearly ineligible (e.g., trials with children or in patients on psychotherapy). Full copies of all remaining articles were obtained. Two independent reviewers checked whether trials met inclusion criteria using a special form. Disagreements were resolved by discussion. The two independent reviewers extracted the following information using a pre-tested form. Methodological quality was assessed using the risk of bias method specified by the Cochrane Handbook based on adequate sequence generation, allocation concealment, blinding, incomplete outcome data, free of selective reporting and free of other bias. The Cochrane Collaboration’s recommended tool for assessing risk of bias is neither a scale nor a checklist. It is a domain-based evaluation in which critical assessments are made separately for different domains. Each domain includes one or more specific entries in a “Risk of bias”. Each pre-specified question about the adequacy of the study in relation to the entry, such that a judgement of “Yes” indicates low risk of bias, “No” indicates high risk of bias and “Unclear” indicates unclear or unknown risk of bias. These are plotted in a graphical format in terms of the fraction of studies in each level of quality [Figure 1].

The only outcome reported in most of the papers was 4 STAXI scale scores and, in one study, the symptom scores were also reported [Table 1]. The summary statistics used for metaanalysis of continuous data, which used the same scales, are the mean differences. In this approach, the standard deviations are used together with the sample sizes to compute the weight given to each study. Studies with small standard deviations are given relatively higher weightage while studies with larger standard deviations are given relatively lower weightage. Using RevMan 5, we calculated the weighted mean differences for 4 STAXI scale scores. We tried to calculate effect sizes for single trials as often as possible and we noted that the group score differences only were having wide standard deviations and not the estimates themselves. Hence, we calculated the fixed-effect estimates and heterogeneity was assessed using the Chi-square test of heterogeneity along with visual inspection of the graph. This Chi-squared test is included in the forest plots in RevMan outputs. It assessed whether observed differences in results are compatible with chance alone. A low P-value (or a large Chi-squared statistic relative to its degree of freedom) provided evidence of heterogeneity of intervention effects (variation in effect estimates beyond chance). A significance level <0.10 was interpreted as evidence of heterogeneity. I ^2^ statistics provided the percentage of the variability in effect estimates due to heterogeneity rather than sampling error (chance). The significance of the summary effects of the metaanalysis was calculated using Z test of the null hypothesis. In this case, *P*-values <0.05 were considered as “statistically significant” and were interpreted as being small enough to justify rejection of the null hypothesis.

**Table 1:** Studies that were included in the analysis

*Study*	*Subjects*	*Diagnosis*	*Year*	*Scale used*	*Duration (weeks)*
Nickel *et al*.[14]	Females	BPD	2004	STAXI	8
Nickel *et al*.[15]	Males	BPD	2005	STAXI	8
Nickel *et al*.[16]	Females	Major depression	2005	STAXI	10
Muehlbacher *et al*.[17]	Both	Chronic low backache	2006	STAXI	10
Loew *et al*.[18]	Females	BPD	2006	Symptom check list (HOS)	10

## Results

We identified 776 studies concerned with the use of anticonvulsants involving anger/aggression measurements. Topiramate featured in 24 studies out of these. We could not include several studies in the review because they were either open labeled or were retrospective analysis of data to assess the therapeutic outcome [Table 2]. Only five studies were randomized controlled trials [Table 3] and hence were included.

**Table 2:** Studies that were excluded from the analysis

Study	Reasons for excluding
Gobbi *et al*.[11]	Retrospective study
Canitano[12]	Open-labeled study
Nickel and Loew[20]	Open-labeled study
Nickel[21]	Open labeled
Nickel[22]	Duplicate study
Kossoff and Pyzik[23]	Open-labeled study performed in children
Fhager *et al*.[24]	Retrospective study
Janowsky *et al*.[25]	Open-labeled retrospective study

**Table 3:** Methodological characteristics of the included studies

*Study*	*Allocation concealment*	*Method*	*Participants*	*Diagnosis*	*Interventions*	*Duration of treatment*	*Outcome*
Nickel *et al*.[14]	Unclear	Double-blind, randomized in a 2:1 ratio, placebo-controlled parallel study	21/31, females 100% Age: 25.5 years	Borderline personality disorder meeting SCID criteria	Topiramate 50–250 mg/day	8 weeks	STAXI scale
Nickel *et al*.[15]	Clear	Double-blind, randomized placebo-controlled parallel study	22/44, females 0% Age: 29.5 years	Borderline personality disorder meeting SCID criteria	Topiramate 50-250 mg/day		8 weeks	STAXI scale
Nickel *et al*.[16]	Unclear	Double-blind, randomized placebo-controlled parallel study	32/64, females 100% Age: 32.5-50.5 years	Major depression meeting SCID criteria	Topiramate 50-200 mg/day	10 weeks	STAXI scale
Muehl-bacher *et al*.[17]	Unclear	Double-blind, randomized placebo-controlled parallel study	48/96, females 30% Age: 43.4-53.2 years	Chronic low back pain of more than 6 months duration	Topiramate 50-300 mg/day		10 weeks	STAXI scale
Loew *et al*.[18]	Clear	Double-blind, randomized placebo-controlled parallel study	28/56, females 100% Age:19.6-30.2 years	Borderline personality disorder meeting SCID criteria	Topiramate 25-200 mg/day		10 weeks	HOS scale

Overall, we got 121 (+28 for HOS score) patients in the topiramate arm and 110 (+28 for HOS score) controls in the placebo arm. The studies varied in terms of inclusion criteria such as BPD, depression and even low back ache. There were separate studies for men and women. The assessment of anger/aggression varied in one of the studies, as given in Table 1.

Nickel[15] and colleagues initiated a randomized controlled trial in 2004, as there were only individual case histories and retrospective studies till then, on the use of topiramate on aggressive states. As the first successful randomized controlled trial on the issue, the study had many advantages. The condition studied was purely BPD in females, as diagnosed by DSM-IV criteria (as per SCID I and SCID II interviews). The study dealt with women aged between 20 and 35 years who were more susceptible to BPD than men and STAXI was used as the primary outcome measure. Twenty-one patients were included under the topiramate arm and 10 were put into the placebo group. The dosage of topiramate was titrated from 50 mg/day to 250 mg/day by the sixth week. The duration of the study was 8 weeks. Two subjects were dropped out of the study in the topiramate arm because they failed to show up for the weekly evaluations.

Males are comparably less affected by BPD and comprise of only 25% of all subjects with BPD. Aggressive impulsivity is the major symptom in BPD. Nickel[16] and colleagues conducted a directed study for BPD in males wherein the same standards as the previous study in females were applied. There were 22 subjects each in the topiramate and placebo arms. Comorbidities were mentioned in the study, such as mood, anxiety, somatoform and eating disorders. Recreational drug use was also present in some of the study subjects. Two subjects were dropped from the study because of irregular weekly evaluation, and this time it was from the placebo arm.

Topiramate was put to test in another randomized controlled trial in 2005 by Nickel[17] and colleagues to female depressive patients. This was a 10-week study, which enrolled 64 subjects, and grouped them into topiramate and placebo arms in a 1:1 ratio. DSM criteria were used to diagnose the condition and Hamilton Depression Rating Scale was applied to objectively measure the depression symptoms. STAXI scales were used as the outcome measure. The analysis was carried out on an intent-to-treat basis. Two subjects from the topiramate group and two from the placebo group were dropped because they failed to show up on two periodic evaluations.

In a significant study by Meuhlbacher[18] on an unrelated condition, i.e. chronic low back pain, topiramate was titrated from 50 mg/day to 300 mg/day in 48 subjects. The effect was compared with a placebo group. Both males and females were part of the study. The dropouts were two in the topiramate group and five in the placebo group. The analysis was carried out on an intent-to-treat basis. The STAXI scores were somewhat lower compared to BPD because the aggression was related to the pain experience and not to any functional maladies.

In another study by Loew *et al*. in 2006,[19] 56 females with BPD were randomized to receive topiramate 50–200 mg/day or placebo in a 1:1 ratio. The primary outcome measures used were symptomatic improvement as on HOS scale. The data were analyzed on an intent-to-treat basis. One patient from the topiramate group and three from the placebo arm, who absented themselves for more than two evaluations, were dropped.

Based on four studies that measured anger on the STAXI scale, we could arrive at a weighted mean difference of −3.16 (−3.64 to −2.68) in State Anger. The reduction in the score was highest in BPD patients as compared to those with low back ache. Trait Anger dropped by −2.93 (−3.49 to −2.37), especially in female BPD patients. Anger-In reduced more or less uniformly across the studies by −1.43 (−1.84 to −1.03). Anger-Out decreased by −2.8 (−3.19 to −2.42). This effect was minimal among the male BPD patients. Anger Control uniformly increased across the four studies by 2.32 (2.00-2.64). Overall, because the studies were conducted in more or less similar conditions, the fixed effects model was adopted for reporting even though some heterogeneity was evident in three of the measurements. The fifth study that was included in the analysis also demonstrated a consistent fall in the hostility score among female BPD patients by −9.30 (−10.85 to −7.75), thereby reinforcing the findings of other studies. Because the STAXI scales were measuring various facets of anger/hostility and we were not sure which component suitably matched the hostility scale, a standardized mean difference analysis was not performed. The only suggestion of non-compliance in this series of studies came from the difference in the number of dropouts in topiramate versus placebo, i.e. the absolute risk reduction, which was 5.34% in favor of topiramate.

From the above findings, it is evident that patients in the topiramate group were significantly benefited, as shown by improvement in symptoms. Also, the dropout rates due to various adverse effects were lower with the topiramate group. This suggests that topiramate is significantly effective in controlling anger/aggression without causing significant adverse effects.

## Discussion

BPD, being a condition that predominantly affects females, our review is generalizable mostly to females from 19 to 53 years of age. Males were represented in two studies, one on BPD and one on low back ache [Table 1]. We did not undertake any subgroup analysis as the proportion of studies with males was less. We were content to report the findings as such with whatever effect size we obtained from individual studies. Altogether, we were satisfied about the balanced publication as per the funnel plots derived and the effect sizes [Table 4] obtained from various individual studies. There are instances in which we have obtained a Chi-square for heterogeneity. However, we have only reported the fixed effect model now. We also did not undertake a standardized mean difference calculation with regard to the hostility subscale for the symptom checklist in one of the studies as we were not sure which of the STAXI constructs fairly represented hostility as such.

**Table 4:** Mean difference of the effects between the topiramate arm and the control arm in various included studies with 95% confidence intervals (in parenthesis)

*Mean difference*	*Diagnosis*	*Subgroup*	*Outcome*	*State Anger*	*Trait Anger*	*Anger-In*	*Anger-Out*	*Anger Control*	*Hostility*
Nickel *et al*.[14]	BPD	Females	STAXI	−6.50 (−7.78, −5.22)	−5.60 (−7.03, −4.17)	−2.60 (−5.22, 0.02)	−5.00 (−6.28, −3.72)	2.90 (2.18, 3.62)	
Nickel *et al*.[15]	BPD	Males	STAXI	−5.90 (−7.82, −3.98)	−1.60 (−3.05, −0.15)	−1.00 (−1.55, −0.45)	−1.00 (−1.55, −0.45)	2.50 (1.95, 3.05)	
Nickel *et al*.[16]	Depression	Females	STAXI	−3.10 (−4.22, −1.98)	−2.50 (−3.66, −1.34)	−1.80 (−37.85, 34.25)	−5.10 (−6.36, −3.84)	2.80 (1.89, 3.71)	
Muehlbacher *et al*.[17]	Chronic low back pain	Males and females	STAXI	−2.10 (−2.72, −1.48)	−2.70 (−3.52, −1.88)	−1.90 (−2.51, −1.29)	−4.30 (−4.99, −3.61)	1.60 (1.04, 2.16)	
Loew *et al*.[18]	BPD	Females	HOS						−9.30 (−10.85, −7.75)
Overall				−3.16 (−3.64, −2.68)	−2.93 (−3.49, −2.37)	−1.43 (−1.84, −1.03)	−2.80 (−3.19, −2.42)	2.32 (2.00, 2.64)	−9.30 (−10.85, −7.75)

**Figure 2:**
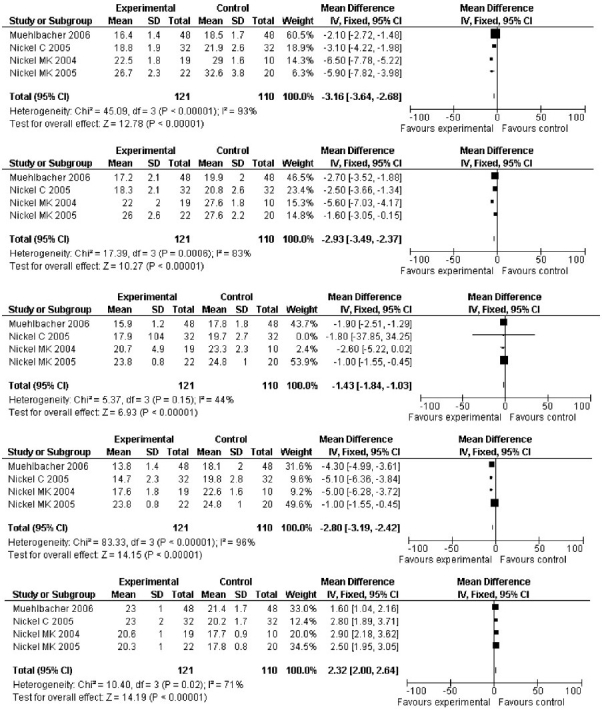
Forest plots and other analysis results of effect sizes as per State Anger, Trait Anger, Anger-In, Anger-Out and Anger Control (STAXI) measurements in topiramate versus placebo trials.

With a fairly good quality of studies in the analysis, the study came to a conclusion that there is sufficient evidence to suggest that topiramate is significantly efficient in stabilizing the “Trait Anger” while reducing the “State Anger” [Figure 2]. “Anger-Out” and “hostility” were reduced significantly. “Anger-In” was the feature that was least affected, although this was significant. As mentioned in results, we could get 121 (+28 for hostility score) patients in the topiramate arm and 110 (+28 for hostility score) controls assigned to the placebo arm, although studies varied in terms of inclusion criteria such as BPD, depression and even low back ache. Five patients were dropped from the topiramate arm and 12 patients were dropped from the placebo arm. Most of the dropouts were due to irregular follow-up than due to psychological adverse events. There was no suggestion of topiramate precipitating psychomorbidity, as was cited by Mula *et al*.[26] in 2003. 8.70 percent of the control subjects probably had some mild adverse outcome as they dropped out of the study, whereas only 3.36% of the experimental subjects had a similar adverse outcome and did the same. The difference, i.e. the absolute risk reduction, was 5.34%, in favor of topiramate. The Number Needed to Treat was thus 19. This means that about one in every 19 patients will benefit from the topiramate treatment without non-compliance.

## Conclusion

Topiramate appears to be a safe and effective drug in the management of anger/aggression. This systematic review has many limitations. First, the sample size was relatively small and the percentage of males included is less compared to that of females. The study duration was generally only 8–10 weeks, which reduced the incidence of adverse effects and the dropout rate. Additional research involving long-term studies is needed to determine whether these results can be reproduced and how long-lasting are the benefits of topiramate in anger control.
